# Exploring co-occurring conditions in Iraqi children with autism spectrum disorder: prevalence, characteristics, and potential risk factors

**DOI:** 10.3389/fpsyt.2025.1592374

**Published:** 2025-07-11

**Authors:** Ahmed Kamil Toman, Hulla Raoof AbdulRasool, Faris Lami, Shatha Mohammed Jasim, Osamah Abbas Jaber, Nahid Dehghan Nayeri, Mahdi Shafiee Sabet, Ghaith Al-Gburi

**Affiliations:** ^1^ Al-Subtain Academy for Autism and Neurodevelopmental Disorders, Karbala, Iraq; ^2^ Baghdad Teaching Hospital, Baghdad, Iraq; ^3^ Imam Hussein Center for Autism, Babil, Iraq; ^4^ Al-Subtain University of Medical Sciences, International Branch of Tehran University of Medical Sciences, Karbala, Iraq; ^5^ Department of Community Medicine, College of Medicine, University of Baghdad, Baghdad, Iraq; ^6^ Nursing and Midwifery Care Research Centre, School of Nursing and Midwifery, Tehran University of Medical Sciences, Tehran, Iran; ^7^ Department of Family Medicine, School of Medicine, Tehran University of Medical Sciences, Tehran, Iran; ^8^ School of Biosciences, Birmingham University, Birmingham, United Kingdom

**Keywords:** autism, medical co-occurring conditions, epilepsy, obesity, underweight, sleep

## Abstract

**Introduction:**

Co-occurring conditions are common in children with autism spectrum disorder (ASD) and have important negative impacts on the children and their families. For Iraqi children, local healthcare systems tend to place more emphasis on the management of ASD itself while neglecting co-occurring conditions.

**Objectives:**

This study aims to investigate the prevalence, characteristics, and potential risk factors of co-occurring epilepsy, sleep, and weight issues among Iraqi children with ASD.

**Methods:**

A multicenter cross-sectional study was conducted from January 24 to August 7, 2024, including children from Imam Hussein Centre, Al-Subtain Academy for Autism and Neurodevelopmental Disorders, and Baghdad’s National Centre for Autism and Child Psychiatry. A structured questionnaire was used, including 35 items for demographic information, epilepsy, sleep problems, and weight issues.

**Results:**

Our sample included 240 children, of whom 34 (14.2%) had co-occurring epilepsy, 178 (74.2%) had at least one sleep problem, and 104 (43.3%) were obese. Among children with epilepsy, 18 (52.9%) received their diagnosis before ASD. The most prescribed anticonvulsant, sodium valproate, was noted in 18 (52.9%) cases. Difficulty falling asleep was the most common sleep problem, affecting 97 (40.4%), while sleepwalking was reported in only 26 (10.8%). Significant differences in the body-mass index were observed based on risperidone use (adjusted p-value = 0.036, R-value = 0.163, 95% CI: 0.031, 0.288), sleep duration (r = -0.166, adjusted p-value = 0.036), and diet (adjusted p-value = 0.036, ϵ^2^ = 0.038, 95% CI: 0.005, 0.087). However, no significant association was demonstrated between BMI and screen time (adjusted p-value = 0.264).

**Conclusion:**

Co-occurring conditions are common among children with ASD and should be assessed simultaneously. Additionally, since some of the children might be diagnosed with epilepsy first, it is important to consider co-occurring ASD in their diagnosis.

## Introduction

The Diagnostic and Statistical Manual of Mental Disorders 5 (DSM-5) defines autism as a neurodevelopmental condition with two major criteria: (A) social and communication difficulties, and (B) restrictive and repetitive behaviors and interests. In 2023, the American Center for Disease Control estimated that around 1 in 36 children (2.8%) might have ASD, while estimates from West Asia have ranged from 0.07% to 1.80%, as such, concerns have been raised about under-diagnosis and under-reporting in the region (1, 2).

It is acknowledged that both verbal and nonverbal communication may be affected and that within the criteria for repetitive and restrictive behaviors, various patterns have been identified, such as stereotypes (motor, speech, use of objects), insistence on sameness, fixated interests, and hypo- or hyperreactivity to sensory input (3, 4). These children are also more likely to have psychiatric and somatic co-occurring conditions, which complicates the diagnosis and makes it challenging to accurately estimate the prevalence. Previous studies have shown that compared to their siblings, children with ASD have an increased prevalence of psychiatric conditions (ADHD, sleep disorders, anxiety, and depression), neurological conditions (epilepsy, motor disturbances, headache disorders), and other somatic conditions, such as gastrointestinal (GIT) disturbances (5, 6). These comorbid conditions might occur due to shared risk factors with ASD, including both genetic and perinatal brain insults (7). Additionally, psychosocial difficulties in children with ASD might contribute to the development of comorbid depression and anxiety (8). Collectively, co-occurring conditions are present in up to 74% of individuals with ASD and are associated with increased parenting stress and reduced quality of life among these children (7, 9, 10). Diagnostic difficulties can also occur as features of co-occurring conditions might sometimes mimic those of autism; food selectivity, as an example, could be attributed to an insistence on sameness as a feature of autism itself or a consequence of food allergies and other GIT disorders (11).

Epilepsy is considered one of the most important neurological co-occurring conditions of autism, with a prevalence ranging from 9% to 19%, based on whether population-based or clinic-based samples are selected (12). Children with co-occurring epilepsy might present with different types of seizures without a specific type showing a clear predominance (13, 14). Additionally, they are more likely to suffer from intellectual issues and severe forms of autism (15, 16). Treatment issues might also arise due to the possible effect of anticonvulsants on symptoms.

Sometimes, the complicated interaction between autism and the co-occurring condition may add to the burden. For instance, 50% to 80% of children with ASD experience sleep disturbances, presumably due to a complex interaction between behavioral issues and biological dysfunctions, such as reduced melatonin production (17, 18). In turn, sleep deprivation has been associated with more severe behavioral profiles, including higher rates of internalizing, externalizing, and repetitive and restrictive behaviors (19). In other cases, clinically important outcomes can result from the interaction of co-occurring conditions with each other. For example, weight gain might be caused by the effects of atypical antipsychotics used to treat irritability but can also be influenced by other co-occurring conditions such as sleep disturbances and GIT disorders (5, 20–22).

Studies have shown that children with these co-occurring conditions suffer from more severe behavioral profiles, tend to have worse adaptive outcomes, and place a higher psychological stress on their families (10, 19, 23). Additionally, the heterogeneity of the prevalence estimates described in the literature has been attributed to differences in the sampled populations, which re-enforces the necessity of conducting local surveys to better understand the scope of the problem and guide the allocation of healthcare services for children affected by co-occurring conditions.

In Iraq, similar to other lower- and middle-income countries, several obstacles hinder the delivery of appropriate care for children with ASD. A field report from the northern region has discussed that these obstacles might occur at different levels, including organizational (social stigma, prioritizing physical illness), professional (resource shortage, inadequate training), and personal (negative provider behaviors) (24). The lack of culturally valid assessment tools has also been cited as a barrier against ensuring that children with ASD receive treatment in a timely manner (25). According to expert opinions, the three co-occurring conditions of epilepsy, sleep disturbances, obesity or underweight tend to be neglected in the management of Iraqi children with ASD in clinical settings, despite being more straightforward to assess in these children compared to other co-occurring conditions like depression, anxiety, and headache disorders. From a research point of view, we were able to identify two studies related to the prevalence of abnormal Electroencephalogram (EEG) in non-epileptic children with ASD and one including children with co-occurring epilepsy (16, 26, 27). However, none that address the characteristic and potential risk factors of epilepsy in these children. We were also able to identify two studies reporting the prevalence of obesity but not the prevalence of potential risk factors (28, 29). Additionally, a previous study estimated the prevalence of sleep problems but not the treatment used by the parents or the association between sleep duration and the body mass index (BMI) in these children (30).

As such, the primary aim of this study was to investigate the prevalence and characteristics of co-occurring epilepsy, sleep disturbances, and weight issues in Iraqi children with ASD. The prevalence of different potential risk factors for these conditions was also assessed as a secondary aim.

## Materials and methods

### Study design and setting

A descriptive multi-center cross-sectional survey was conducted as a part of a research collaboration between Al-Subtain University of Medical Sciences and Al-Subtain Academy for Autism and Neurodevelopmental Disorders, from January 24 to August 7, 2024. Children with ASD were selected from three specialized healthcare centers distributed across three different provinces: Al-Subtain Academy in Karbala, Imam Hussein Centre for Autism in Babil, and Baghdad’s National Centre for Autism and Child Psychiatry. Within their provinces, these centers are the only ones responsible for providing specialized care for these children, and collectively, they are responsible for providing care to most of the children with ASD from the middle and southern regions of Iraq. Data was collected from these healthcare centers between April 1 and June 1, 2024.

### Eligibility criteria

Children with a confirmed diagnosis of ASD were recruited from the pediatric age groups; defined by the Iraqi Ministry of Health and Environment as an individual <18 years of age. At each healthcare center, to confirm the diagnosis, two consultants in children and adolescent psychiatry had to agree on a diagnosis of ASD, as defined in the DSM-5, through an evaluation consisting of both parent’s interview and an observation of the child’s behaviors (4). As per local professional training requirements, a consultant is defined as a healthcare professional with at least 10 years of experience in general psychiatry and at least 5 years of experience in their selected subspeciality (children and adolescent psychiatry).

### Data collection tool

A structured questionnaire divided into five sections was used for data collection (see research questionnaire). The first section included items related to the child’s demographic information, such as current age, gender, age at diagnosis with autism, and family history of autism. Thirty-five items were selected to assess co-occurring conditions, following a review of previous studies (31–35), and subsequently arranged across four sections: epilepsy (10 items), sleep disturbances (12 items), and weight issues (9 items).

For epilepsy, five items were used to address characteristics such as age of onset, type of seizures, treatment, and control status in the last six months. Children with controlled epilepsy were defined as those who had no episode of seizure in the last six months. The remaining six items focused on potential risk factors, including family history of neurological conditions (epilepsy, intellectual disability, etc.), history of meningitis or encephalitis, previous head trauma, and perinatal factors. Each item was presented as a yes/no question, with some items requiring additional information, like the age of onset of epilepsy and the timing of previous head trauma and meningitis/encephalitis.

Sleep quantity was investigated by enquiring about sleep duration during the night (in hours), while sleep quality was assessed by enquiring about the presence of ten different sleep problems, including difficulty falling asleep, parasomnias (such as sleepwalking), waking up during the early morning, among other behaviors (see research questionnaire). Parents were also asked whether they have used any medications to manage sleep problems in their children and the type of medications.

In the fourth section, the child’s current weight and height were collected to calculate the BMI in kg/m^2^. Eight additional items were also included as potential risk factors: diet, previous weight changes, history of chronic conditions, type of medications used for chronic conditions, daytime activity, self-reported family lifestyle, and daily screen time. Diet was classified into three categories: a low-calorie diet of fruits and vegetables, a high-calorie diet (consisting mostly of snacks and high-carbohydrate foods like grains and pastry), and a balanced diet.

### Data collection procedure

Pediatricians with experience in working with autism interviewed the parents using the previously discussed questionnaire. Each interview took around 20–30 minutes were the physician recorded the child’s demographic information and enquired about the presence of epilepsy. If epilepsy was not present, questions related to epilepsy’s characteristics and risk factors would not be asked. Other questions were administered to all participants.

### Data quality reassurance

First, data was double-checked to ensure that no errors were introduced when converting paper records into an electronic dataset. Second, the presence of epilepsy had to be confirmed by medical records provided by the parents, including EEG records, physicians’ notes, and prescriptions. Initially, assessing the presence of co-occurring ADHD was also included as an aim in this study. However, during the data collection period, medical records including a psychiatrist- or a pediatrician-level diagnosis of ASD could not be acquired in many cases. As such, co-occurring ADHD was omitted from this study. Third, confirmatory bias was reduced at the level of data collection by delaying any instances of statistical analysis until the end of data collection. Finally, reporting was conducted according to the STROBE guidelines described by Equator Network for cross-sectional to reduce reporting bias (36).

### Statistical analysis

Descriptive statistics were conducted using The Statistical Package for Social Sciences (SPSS) version 28, while the R Language and Environment for Statistical Programming ver. 4.4.2 and its associated packages were utilized for hypothesis testing and effect size estimation (see the Data and Material Availability Statement) (37–40).

Normality was assessed using the Shapiro-Wilk test, which revealed non-normal distributions for all the continuous variables in the study (**Table 1** **).** The D’Agostino-Pearson K^2^ test was then conducted as a normality test that is less affected by large sample sizes and revealed similar results (**Table 1**) (41). Therefore, continuous variables were summarized using the median and interquartile range (IQR) and hypothesis testing was conducted using nonparametric tests.

**Table 1:** Normality testing for the study variables (N = 240).

Variables	Shapiro-Wilk			D’Agostino-Pearson K^2^	
	Statistic	df	P-value	Statistic	P-value
Age (Years)	0.910	240	**<0.001**	49.113	**<0.001**
Age of diagnosis with autism (Years)	0.911	240	**<0.001**	22.54	**<0.001**
Night sleep duration (Hours)	0.952	240	**<0.001**	11.177	**0.002**
BMI (kg/m^2^)	0.909	240	**<0.001**	50.660	**<0.001**
Daily screen time (Hours)	0.907	240	**<0.001**	47.767	**<0.001**

Bold fond indicates statistically significant differences.

Categorical variables were presented as counts and percentiles. BMI was then classified using the PediTool electronic calculator based on the American Academy of Paediatrics guidelines, and the CDC growth charts for children between 2 and 20 years (42–44). Children were also grouped based on their daily screen time following the American Academy of Child and Adolescent Psychiatry’s recommendations (45).

Sample characteristics were compared between the three healthcare centers involved during data collection. The Chi-square and Krustkal Wallis tests were utilized for this purpose and revealed no statistically significant differences between the three samples, thereby supporting data pooling during further analysis. The distribution of BMI classification could not be compared between the three samples due to insufficient subgroup sample sizes. The associations between BMI and potential risk factors were also assessed using Kruskal-Wallis and Man-Whitney U tests, while Spearman’s rank correlation was used to examine the correlation with sleep duration and daily screen time. For all hypotheses testing, 0.05 was selected as a cut-off point for statistical significance. The Benjamin-Hochberg procedure was also used to adjust p-values for multiple testing and reduce the false discovery rate to 5% (46).

Effect size point estimates were calculated based on the utilized hypothesis test; Krustkal Wallis test (Epsilon squared), Man-Whitney U test (R-values), Chi-square test (Cramer’s V). For all point estimates, the 95% confidence interval was also calculated using bias-corrected and accelerated bootstrapping with 10000 simulated samples. For Epsilon square, a value of ≥0.01 indicated a small effect size, ≥0.06 for moderate, and ≥0.14 for large effect size (47). For R-values, a value of ≥0.1 indicated a small, ≥0.3 for moderate, and ≥0.5 for large effect size (48). For Cramer’s V, a six-level scale was used [negligible: <0.10, weak: ≥0.10, moderate: ≥0.20, relatively strong: ≥0.40, strong: ≥0.60, very strong: ≥0.80] (49).

## Results

### Sample characteristics

Our sample included 240 children, of whom 136 (56.7%) were from Al-Subtain Academy, 67 (27.9%) from Imam Hussein Centre, and 37 (15.4%) from Baghdad’s National Center. As shown in **Table 2**, 34 (14.2%) had co-occurring epilepsy, 178 (74.2%) had at least one of the listed sleep problems, and 104 (43.3%) were obese.

**Table 2:** Sample characteristics across the three healthcare centers (N = 240).

Sample characteristics	Al-Subtain Academy n = 136	Imam Hussein Centre n = 67	Baghdad’s National Centre n = 37	Total N=240	p-value	Effect size (95% CI)^d^
Age (Years) (Median, IQR)	5 (4, 7)	5 (4.25, 6.5)	5 (4, 6.5)	5 (4, 6.5)	0.655^b^	0.004 (<0.001, 0.015)^e^
Gender (N, %) Male Female						
	101 (74.3)	49 (73.1)	28 (75.7))	178 (74.2)	0.960^c^	0.018
	35 (25.7)	18 (26.9)	9 (24.3)	62 (25.8)		(<0.001, 0.022)^f^
Age at ASD diagnosis (Median, IQR)	3 (2 – 3)	3 (2 – 3.5)	3 (2 – 3)	3 (2, 3)	0.273^b^	0.011 (<0.001, 0.039)^e^
Family history of ASD (N, %)	41 (30.1)	23 (21.8)	14 (12.0)	78 (32.5)	0.629^c^	0.062 (0.002, 0.131)^f^
Presence of comorbid epilepsy (N, %)	16 (11.8)	12 (17.9)	6 (16.2)	34 (14.2)	0.451^c^	0.080 (0.005, 0.166)^f^
Presence of at least one sleep problem (N, %)	105 (77.2)	51 (76.1)	22 (59.5)	178 (74.2)	0.083^c^	0.143 (0.024, 0.267)^f^
BMI (kg/m^2^) (Median, IQR)	18.2 (15.8, 22.5)	17.9 (14.9, 20.9)	17.6 (16.0, 20.8)	18.0 (15.7, 21.9)	0.374^b^	0.008 (<0.001, 0.033)^e^
BMI classification^a^ (N, %)						
Underweight (<5^th^ percentile)	15 (11.0)	5 (7.5)	2 (5.4)	22 (9.2)	N/A	N/A
Normal weight (5^th^ – <84^th^ percentile)	43 (31.6)	26 (38.8)	14 (37.8)	83 (34.6)		
Overweight (85^th^ - <94^th^ percentile)	12 (8.8)	11 (16.4)	8 (21.6)	31 (12.9)		
Obesity class I (≥95^th^ percentile)	28 (20.6)	12 (17.9)	7 (18.9)	47 (19.6)		
Obesity class II (95^th^ percentile: 120% - 140%)	12 (8.8)	10 (14.9)	4 (10.8)	26 (10.8)		
Obesity class III (95^th^ percentile: >140%)	26 (19.1)	3 (4.5)	2 (5.4)	31 (19.9)		

a BMI was classified according to the American Academy of Paediatrics guidelines based on the CDC growth charts for children between 2 and 20 years.

b Kruskal-Wallis test was used for analysis with 0.05 as the cut-off point for statistical significance.

c Chi-square test was used for analysis with 0.05 as the cut-off point for statistical significance.

d 95% confidence intervals were calculated using bias-corrected and accelerated bootstrapping with 10000 simulated samples.

e Epsilon-squared was used to assess the effect size with ≥0.01 for small, ≥0.06 for moderate, and ≥0.14 for large effect sizes.

f Cramer’s V was used to assess the effect size with <0.10 for negligible, ≥0.10 weak, ≥0.20 moderate, ≥0.40 relatively strong, ≥0.60 strong, ≥0.80 for very strong effect size.

### Epilepsy among children with ASD

Of the 34 children with co-occurring epilepsy, 18 (52.9%) were diagnosed with epilepsy before autism. In terms of characteristics, most of the children had generalized tonic-clonic seizures (33, 97.1%) (**Table 3**). 12 (35.3%) of those with epilepsy had a family history of neurological conditions, including epilepsy (8 children), febrile seizure (3 children), and 1 child had a family history of both epilepsy and intellectual issues.

**Table 3:** Characteristics of co-occurring epilepsy in children with autism spectrum disorder (N = 34).

Characteristic	Distribution
Age of onset (Years) (Median, IQR)	2.25 (1.63, 3)
Timing relative to autism diagnosis (N, %)	
Before autism diagnosis	18 (52.9)
Within the same year	3 (8.8)
After autism diagnosis	13 (38.2)
Type of seizures (N, %)	
Generalized tonic-clonic	33 (97.1)
Focal	1 (2.9)
Controlled in the last 6 months (N, %)^a^	15 (44.1)
Family history of neurological conditions or epilepsy (N, %)	12 (35.3)
History of meningitis or brain infection (N, %)	5 (14.7)
Timing of infection relative to epilepsy onset (N, %)^b^	
Before epilepsy	4 (80.0)
After epilepsy	1 (20.0)
History of head trauma (N, %)	9 (26.5)
Timing of head trauma relative to epilepsy onset (N, %)^b^	
Before epilepsy onset	1 (11.1)
Within the same year	2 (22.2)
After epilepsy onset	6 (66.7)
Obstructed labor (N, %)	11 (32.4)
NICU admission >5 days (N, %)	11 (32.4)
Ventilation requirement during neonatal life (N, %)	10 (29.4)
Usage of anti-epileptic medications (N, %)^c^	
Sodium valproate	18 (52.9)
Levetiracetam	1 (2.9)
Topiramate	2 (5.9)
Oxcarbazepine	4 (11.8)
Diazepam	2 (5.9)
Carbamazepine	9 (26.5)

a Children with controlled epilepsy were defined as those who had no episode of seizure in the last six months.

b The number of children with a history of infection and head trauma was selected as the denominator for these variables respectively.

c Categories might not add up to 100% as some children might be on more than one anti-epileptic medication.

### Sleep problems among children with ASD

Difficulty falling asleep was the most common issue, affecting 97 (40.4%) of our sample, followed by sleep-onset-associated problems, which affected 78 (32.5%) (**Table 4**). The least commonly reported problems were sleepwalking and daytime sleepiness, affecting 26 (10.8%) and 17 (7.1%) of children, respectively.

**Table 4:** Characteristics of sleep problems among children with autism spectrum disorder (N = 240).

Characteristic	Distribution
Difficulty falling asleep (N, %)	97 (40.4)
Sleepwalking (N, %)	26 (10.8)
Waking up during the early morning (N, %)	75 (31.3)
Problems arising during the morning (N, %)	34 (14.2)
Day-time sleepiness (N, %)	17 (7.1)
Sleep-onset associated problems^a^ (N, %)	78 (32.5)
Difficulty breathing or snoring during sleep (N, %)	40 (16.7)
Teeth grinding (N, %)	29 (12.1)
Confusional arousal (N, %)	34 (14.2)
Behavioral disorders during sleep (N, %)	46 (19.2)
Type of behavioral disorder^b^ (N, %)	
Behavioral/Vocalization	19 (41.3)
Motor	24 (52.2)
Night sleep duration (Hours) (Median. IQR)	8 (7, 9)
Usage of sleep medications (N, %)	
Melatonin	41 (17.1)
Chlorpheniramine	1 (0.4)
Imipramine	1 (0.4)

a Sleep-onset problems e.g. inability to sleep without toys, pets, or parents.

b The number of children with behavioral sleep disorder was selected as the denominator, with 3 missing values.

### Weight issues among children with ASD

**Table 5** summarizes the characteristics and potential risk factors for weight issues in children with ASD. Accordingly, 38 (15.8%) had a high-calorie diet, 48 (20%) were inactive, and 29 (12.1%) had chronic conditions, including asthma (7 children), diabetes mellitus type 1 (4 children), and psoriasis (3 children), among other conditions. Additionally, 150 (62.5%) of the children in our sample had a daily screen time exceeding the amount recommended by the American Academy of Child and Adolescent Psychiatry.

**Table 5:** Potential factors associated with BMI in children with autism spectrum disorder (N = 240).

Factors	Study groups	Frequency N (%)	BMI kg/m^2^ Median (IQR)	P-value	Adjusted P-value^d^	Effect size (95% CI)^g^
Diet	Low-calorie	85 (35.4)	17.4 (15.3, 20.2)	0.011^b^	**0.036**	0.038 (0.005, 0.087)^e^
	Balanced	117 (48.8)	18.1 (15.5, 22.4)			
	High-calorie	38 (15.8)	19.2 (17.3, 25.2)			
History of chronic conditions	No	211 (87.9)	18.1 (15.7, 22.2)	0.311^c^	0.350	-0.066 (-0.180, 0.055)^f^
	Yes	29 (12.1)	17.2 (16.3, 20.4)			
Family history of weight disorders	No	226 (94.2)	18.0 (15.6, 21.8)	0.232^c^	0.298	0.078 (-0.057, 0.211)^f^
	Yes	14 (5.8)	20.7 (15.9, 25.3)			
Child’s daytime activity	Inactive	48 (20.0)	17.4 (15.2, 21.0)	0.066^b^	0.119	0.022 (0.001, 0.066)^e^
	Moderate	122 (50.8)	17.6 (15.5, 21.7)			
	Overactive	70 (29.2)	18.3 (16.3, 25.0)			
Self-reported Family lifestyle	Inactive	100 (41.7)	18.0 (15.4, 21.4)	0.874^b^	0.874	0.001 (<0.001, 0.004)^e^
	Balanced	95 (39.6)	18.0 (15.8, 22.2)			
	Overactive	45 (18.8)	18.0 (16.5, 22.3)			
Excessive screen time ^a^	No	90 (37.5)	17.3 (15.3, 21.5)	0.176^c^	0.264	0.087 (-0.042, 0.211)^f^
	Yes	150 (62.5)	18.2 (16.0, 22.4)			
Medications: Risperidone	No	210 (87.5)	17.8 (15.5, 21.7)	**0.012**^**c**^	**0.036**	**0.163 (0.031, 0.288)**^**f**^
	Yes	30 (12.5)	20.5 (17.2, 26.7)			

a Classified according to the American Academy of Child and Adolescent Psychiatry recommendations.

b Kruskal-Wallis test was used for analysis with 0.05 as the cut-off point for statistical significance.

c Man-Whitney U test was used for analysis with 0.05 as the cut-off point for statistical significance.

d Bejamin-Hochberg procedure at a false discovery rate of 0.05 was used to adjust p-values for multiple testing.

e Epsilon-squared was used to assess the effect size with ≥0.01 small, ≥0.06 for moderate, and ≥0.14 for large effect sizes.

f R-value was used to assess the effect size with ≥0.1 for small, ≥0.3 for moderate, and ≥0.5 for large effect sizes.

g 95% confidence intervals were calculated using bias-corrected and accelerated bootstrapping with 10000 simulated samples.

Bold fond indicates statistically significant differences.

The use of risperidone had a statistically significant association with BMI, with a small effect size (adjusted p-value = 0.036, R-value = 0.163, 95% CI: 0.031, 0.288) (**Table 5**). Additionally, we found a statistically significant very weak negative correlation between BMI and sleep duration (r = -0.166, adjusted p-value = 0.036). However, no statistically significant correlation was found with daily screen time after adjusting for multiple testing (r = 0.140, adjusted p-value = 0.0068). Additionally, no significant associated was demonstrated between BMI and screen time, when the American Academy of Child and Adolescent Psychiatry classification is considered (adjusted p-value = 0.264) (**Table 5**).

## Discussion

### Epilepsy

Previous reports have estimated the prevalence of co-occurring epilepsy to be in the range of 9% to 19%, noting that clinic-based samples tend to overestimate (12, 35). In this study, we found that the prevalence was 14.2% in a clinic-based sample of 245 children with ASD, similar to the 13.7% figure reported in a 2024 study from Hilla city (27). No official estimate has been reported for the prevalence of epilepsy among Iraqi children, which affects the ability to establish precise comparisons. However, according to data from the Iraqi department of Health and Vital Statistics, by 2016, epilepsy was prevalent among 2.52% of the Iraqi population, indicating that Iraqi children with ASD might suffer from higher rates of epilepsy (50). This, in addition to the fact that around half of these children had uncontrolled epilepsy, points out the necessity to spend more comprehensive efforts to address co-occurring epilepsy, especially, since previous studies have demonstrated that early uncontrolled epilepsy can lead to worse cognitive outcomes and further delay in language acquisition (51–53).

Heterogeneity in the reported estimates has been attributed to the characteristics of the samples, particularly the child’s gender, age, and the presence of intellectual issues (12). In our sample, there was no difference in the presence of co-occurring epilepsy according to gender and no association with the child’s age, presumably because only 2% of the children were above 6 years of age.

Epilepsy was diagnosed before autism in around half of the cases, supporting the imperative of screening for neurodevelopmental conditions among children with epilepsy since they have more than ten-fold the risk of having autism (54).

No specific type of seizure has shown clear predominance in children with co-occurring epilepsy. A study from Bangladesh in 2022 has shown that focal seizures were the most common in these children (51.5%) (13), while another study from India showed that tonic-clonic seizures were the most common (48%), followed by focal impaired awareness seizures (17%) (14). In both the studies mentioned, figures for the prevalence of generalized tonic-clonic seizures were lower than the 97.1% figure mentioned in our results. Compared to the tonic-clonic movements in generalized epilepsy, focal seizures tend to be more subtle; hence, parents might be more likely to seek medical attention when their child experiences generalized seizures. Additionally, focal impaired awareness seizures might present with “automatism”—repetitive movements of the face (e.g., chewing, lip-smacking, etc.) or the limbs (55), which might be mistaken by the parents as repetitive behaviors of autism, leading to underreporting. For these reasons, physicians should be instructed to carefully evaluate epilepsy in children with ASD and note that repetitive behaviors are more persistent and do not cause loss of awareness, unlike automatism, which is more sudden. On an organizational level, institutions responsible for providing care for children with ASD should consider the development and administration of clinical training modules for differentiating epileptic automatism from ASD stereotypies. Video-based learning might be particularly useful in enhancing physicians’ ability to spot differences between the two conditions (56).

The etiology of the co-occurrence between epilepsy and autism has been extensively discussed in the literature, with a focus on underlying neurobiological factors, including shared genetics, intellectual disability, and shared prenatal/postnatal brain insults (7). In our study, approximately one-third of the children had a positive family history of neurological conditions, prolonged admission to the neonatal intensive care unit, or ventilation during early neonatal life. In addition, around one-fifth had a history of head trauma occurring before or within the same year of being diagnosed with epilepsy.

### Sleep

Sleep problems occur in <50% of typically developing children and are more common in children with ASD, with estimates ranging from 50% to 80% (57). In our sample, 74.2% had at least one of the listed sleep problems. Difficulty falling asleep was the most common, consistent with a previous study from Iraq, during which children with ASD scored 2.2 out of 5 (44%) for difficulty in falling asleep (30). Studies have shown that compared to typically developing peers, these children tend to sleep 32.8 minutes less per night and have a sleep latency that is 10.9 minutes longer (58). It has been hypothesized that reduced production of melatonin, as indicated by reduced excretion of urinary metabolites, and changes in GABAergic sleep-promoting areas of the brain are responsible for these sleep disturbances (17, 59). As such, melatonin has been tried and shown success in improving sleep for these children and is being used by 17.1% of the parents in our study.

Behavioral insomnia might also occur when children associate certain objects, people, or events with sleep, leading to an inability to initiate or re-initiate sleep when these conditions are not met (57). This is known as sleep-onset-associated problems and was present in 32.5% of our sample. Pathogenically, these behaviors reflect rituals, as described in the DSM-V, and highlight the importance of sleep hygiene and bedtime routines in the management of sleep problems in addition to pharmacotherapy (4, 33). A previous study has shown that even a few sessions of parents’ sleep education could result in a lower sleep onset delay in these children (60). Based on our results, sleep onset associated problems are common in Iraqi children with ASD, and as such, sleep hygiene education should be included as an instrumental part of parent training and education sessions organized by institutions responsible for providing care for these children.

Parasomnia, including non-REM (confusional arousal, teeth grinding, and sleepwalking) and REM sleep behaviors (both vocalization and motor), might also occur in increased prevalence compared to typically developing children. Sleep-disorder breathing, on the other hand, has been found in similar prevalence in some studies and higher in others, but is still clinically important as it might result in behavioral profiles similar to ADHD. This is a problem, as ADHD is a common co-occurring condition in children with ASD to start with (57, 61, 62).In our study, 16.7% of the children had trouble breathing or snoring during their sleep.

### Weight

According to the WHO country nutrition profile, the prevalence of obesity among Iraqi children between 5–18 years of age was 14.4% in 2016 (63). In our study, children had a median BMI of 18.0 kg/m^2^ [IQR: 15.7 - 21.9], similar to a study from Hilla city, where a mean BMI of 17.6 kg/m^2^ [SD: ± 4.1] was reported (28). Nonetheless, discrepancies are evident in the reported prevalence of obesity, as estimates rely on different classification guidelines. Our study utilized the American Academy of Paediatrics guideline, yielding an obesity prevalence of 42.5%. The previously mentioned study employed a more conservative method, utilizing the 97th percentile of the WHO age and sex-specific growth charts, which yielded an obesity prevalence of 14.7%. A 2024 study from Baghdad recruited 80 children with ASD and reported a 5% estimate for obesity; defined as >95^th^ percentile on the WHO age and sex-matched growth charts (29). The fact that the latter study reported a lower prevalence of obesity than figures from Hilla, despite using a less conservative cut-off on the same growth charts, highlights that differences in estimates across studies come not only from utilizing different classification guidelines but might also arise from differences between the sampled populations or the effects of convenient sampling. Indices of central tendencies and dispersion were not reported for data from Baghdad. Therefore, it is not possible to assess whether the magnitude of difference from our result prior to classification.

Conversely, studies have indicated that children with ASD are also at a higher risk of being underweight (64). The prevalence of being underweight is not reported for the general pediatric population in Iraq, thereby, limiting the potential for informed comparisons. However, the WHO estimates that, in 2018, 2.5% of Iraqi children <5 years had BMI values below the <5^th^ percentile (63). Similarly, a previous study from Hilla city also reported that 2.4% of preschool children were underweight (65). Both figures are lower than the 9.2% reported in our study and the 17.5% rate reported among children with ASD in Baghdad (29).

Altered sensory processing is more common in children with neurodevelopmental conditions and has been connected to food selectivity (66). However, the role of feeding behaviors in the pathogenesis of weight gain or loss has been found in some studies but not in others (67, 68). Similarly, studies have been inconsistent in reporting higher sedentary behaviors (like using electronics) between autistic and non-autistic children (69). Our study revealed that the association between diet and BMI was significant, although with a small effect size. Meanwhile, screen time did not show statistically significant relations with BMI neither as a continuous variable nor as a dichotomous classification based on the American Academy of Child and Adolescent Psychiatry guideline, after adjusting for multiple testing.

Atypical antipsychotics, such as risperidone and aripiprazole, are frequently used for irritability in children with ASD and have been implicated in leptin resistance; an important appetite suppressor inside the body (21). Sleep problems can also contribute to weight gain by decreasing leptin and increasing ghrelin; the latter functions as an appetite promotor (20, 70). In our study, children who used risperidone had statistically significant higher BMI than those who did not. Additionally, a statistically significant negative correlation was detected between night sleep duration and BMI.

### Strength, limitations, and recommendations

This study constitutes the first detailed investigation of the presence, characteristics, and common potential risk factors of co-occurring epilepsy, sleep, and weight issues among Iraqi children with ASD. A multi-centric approach was utilized and involved three specialized healthcare centers responsible for the majority of children ASD from the middle and southern region of Iraq. Effect size measures were reported to enhance the interpretability of the detected statistically significant association.

Despite these strengths, several limitations can be highlighted. Firstly, children were recruited through convenient sampling from healthcare centers in the middle and southern regions of Iraq and might, therefore, not reflect the population of children with ASD on a national level. Specifically, these results might not be generalizable on children from the northern region of Iraq and children whose parents do not present to healthcare services, either due to cultural reasons or inaccessibility (such as parents from rural areas). Secondly, the difference in the prevalence of these co-occurring conditions between children with ASD and typically developing children could not be evaluated due to the absence of an age- and sex-matched comparison group. Thirdly, the prevalence of co-occurring epilepsy, in particular, could not be contextualized due to insufficient information on the presence of intellectual disability and the severity of ASD. Finally, the presence of epilepsy, its type and used medications were established through medical records. However, due to lack of comprehensive electronic health records, the presence of potential risk factors and sleep duration were established with parent-reported data. As such, these items might be subject to recall bias, particularly, perinatal events.

Future studies should be conducted to address these limitations. Specifically, community-based epidemiological surveys should be conducted to include families from varying socioeconomic backgrounds and healthcare accessibility. The inclusion of matched control groups in these surveys will also aid the investigation of excess morbidity among Iraqi children with ASD. More broadly, efforts should be made to establish comprehensive electronic health record systems in these healthcare centers. This baseline data would prove crucial for further research on the follow-up of these children.

## Conclusion

Co-occurring conditions are common among Iraqi children with ASD and should be assessed simultaneously. Clinicians should be aware of the necessity to screen children with epilepsy for autism and that the presentation of focal seizures might be subtle and require careful assessment to differentiate. Parents of children with ASD should be made aware of the importance of nutrition and that certain medications such as risperidone are associated with higher BMI in their children. Sleep problems were presents in the majority of Iraqi children with ASD and should be addressed as they are also associated with higher BMI in these children.

## Data Availability

The R script used for statistical analysis is publicly available on Zenodo at: https://doi.org/10.5281/zenodo.15399111. The dataset used in this study will be made publicly available on Mendeley’s data at: https://doi.org/10.17632/5wbf3cpbyp.1. Further inquiries can be directed to the corresponding author/s.
