# Supplementary material for: Exploring co-occurring conditions in Iraqi children with autism spectrum disorder: prevalence, characteristics, and potential risk factors
Source: Front Psychiatry. 2025 Jul 11;16:1592374. doi: 10.3389/fpsyt.2025.1592374 (PMC12292020; doi:10.3389/fpsyt.2025.1592374)
Supplement: Supplementary file 1 [file Table1.docx]

**Research questionnaire (English version)**

**First:** Demographic factors

| **Child age (years)** |  |
| --- | --- |
| **Gender** | 🞎 Male 🞎 Female |
| **Age at diagnosis with autism (years)** |  |
| **Family history of autism** | 🞎 No 🞎 Yes |

**Second:** Epilepsy

| **Age of onset (years)** |  |
| --- | --- |
| **Type of seizures** |  |
| **Control in the last 6 months** | 🞎 No 🞎 Yes |
| **FHx. of CNS diseases (including epilepsy)** | 🞎 No 🞎 Yes, **Specific:** |
| **Hx. of CNS infection** | 🞎 No 🞎 Yes, **Age:** |
| **Hx. of head trauma** | 🞎 No 🞎 Yes, **Age:** |
| **Hx. of obstructed labor** | 🞎 No 🞎 Yes |
| **Hx. of NICU admission (> 5 days)** | 🞎 No 🞎 Yes |
| **Hx. of ventilation during neonatal life** | 🞎 No 🞎 Yes |
| **Treatment (type & duration)** |  |

**Third:** Sleep problems

| **Difficulty falling asleep** | 🞎 No 🞎 Yes |
| --- | --- |
| **Sleepwalking** | 🞎 No 🞎 Yes |
| **Waking up during the early morning** | 🞎 No 🞎 Yes |
| **Problems arising during the morning** | 🞎 No 🞎 Yes |
| **Day-time sleepiness** | 🞎 No 🞎 Yes |
| **Sleep-onset association problems** e.g. inability to sleep without toys, pets, or parents | 🞎 No 🞎 Yes |
| **Difficulty breathing or snoring during sleep** | 🞎 No 🞎 Yes |
| **Teeth grinding** | 🞎 No 🞎 Yes |
| **Confusional arousal** e.g. fear or confusion when waking up | 🞎 No 🞎 Yes |
| **Behavioural disorder during sleep** | 🞎 No 🞎 Yes **(vocalization or motor)** |
| **Sleep duration during the night (hours)** |  |
| **Treatment for sleep problems** | 🞎 No 🞎 Yes, **specify:** |

**Fourth:** Weight problems

| **Current weight (kg)** |  |
| --- | --- |
| **Current height (cm)** |  |
| **Diet** | 🞎 High-calorie (mostly snacks & carbohydrates)  🞎 Balanced diet  🞎 Low-calorie (mostly fruits & vegetables) |
| **Hx of chronic conditions** | 🞎 No 🞎 Yes, **specify:** |
| **Treatment for chronic conditions** | 🞎 No 🞎 Yes, **specify:** |
| **FHx of weight disorders** | 🞎 No 🞎 Yes **(Underweight or overweight)** |
| **Level of activity during daytime** | 🞎 Inactive 🞎 Moderate 🞎 Overactive |
| **Family lifestyle** | 🞎 Inactive 🞎 Moderate 🞎 Overactive |
| **Daily hours spent on TV, PC, or phone** |  |

**Research questionnaire (Arabic version)**

**معلومات الطفل :**

|  | **عمر الطفل (السنوات)** |
| --- | --- |
| بنت 🞎 ولد 🞎 | **جنس الطفل** |
|  | **العمر عندما تم تشخيصه باضطراب التوحد** |
| كلا 🞎 نعم 🞎 | **هل يوجد احد اخر من العائلة لديه اضطراب التوحد** |

**الصرع :**

|  | **ماهو عمر الطفل عند حدوث اول نوبة صرع (السنوات)** |
| --- | --- |
|  | **نوع الصرع** |
| كلا 🞎 نعم 🞎 | **هل ان الصرع مسيطر عليه في اخر 6 اشهر** |
| نعم, **ما نوع المرض:** 🞎 كلا او لا اعلم 🞎 | **هل لدى العائله امراض عصبيه وراثيه او صرع** |
| نعم, **كم كان عمره:** 🞎 كلا 🞎 | **هل تعرض الطفل لالتهاب السحايا او مرض بكتيري او فايروسي في الدماغ** |
| نعم, **كم كان عمره:** 🞎 كلا 🞎 | **هل تعرض الطفل الى صدمه او ضربه على الرأس** |
| كلا 🞎 نعم 🞎 | **هل كانت ولادته متعسره** |
| كلا 🞎 نعم 🞎 | **هل تم ادخاله الى ردهة الخدج المعقم لاكثر من خمسة ايام** |
| كلا 🞎 نعم 🞎 | **هل تم اجراء تنفس صناعي او تم وضع تنفس صناعي** |
|  | **نوع العلاج ( الجرعه و المده)** |

**مشاكل النوم :**

| كلا 🞎 نعم 🞎 | **لديه صعوبه بالغه عند التوجه للنوم** |
| --- | --- |
| كلا 🞎 نعم 🞎 | **يمشي اثناء النوم** |
| كلا 🞎 نعم 🞎 | **يستيقظ مبكرا** |
| كلا 🞎 نعم 🞎 | **يعاني من صعوبه بالغه عند الاستيقاظ صباحا** |
| كلا 🞎 نعم 🞎 | **ينام اثناء النهار كثيرا** |
| كلا 🞎 نعم 🞎 | **لا ينام الا مع امه او ابيه او العابه او حيوان اليف او اي شئ متعلق به** |
| كلا 🞎 نعم 🞎 | **لديه صعوبة في التنفس او شخير قوي اثناء النوم** |
| كلا 🞎 نعم 🞎 | **صرير الاسنان اثناء النوم** |
| كلا 🞎 نعم 🞎 | **هلع وخوف وتشوش عند الاستيقاض** |
| نعم, **ما نوعها:** 🞎 كلا 🞎 | **اضطرابات سلوكيه اثناء النوم مثل الكلام او حركات لا اراديه** |
|  | **عدد ساعات النوم ليلا** |
| نعم, **ما نوعها:** 🞎 كلا 🞎 | **هل يتناول ادويه خاصه للنوم** |

**مشاكل الوزن**

|  | **الوزن حاليا (بالكيلوغرام)** |
| --- | --- |
|  | **الطول حاليا (بالسنتميتر)** |
| سعرات حراريه عاليه (حلويات و معجنات) 🞎  وسط 🞎  سعرات حراريه قليله (فواكه و خضراوات)🞎 | **نوع الغذاء** |
| نعم, **ما نوع المرض:** 🞎 كلا 🞎 | **هل يعاني الطفل من امراض مزمنه** |
| نعم, **ما نوعها:** 🞎 كلا 🞎 | **هل يتناول ادويه معينه ام لا** |
| نعم, **ما نوع المرض:** 🞎 كلا او لا اعلم 🞎 | **تاريخ مرضي وراثي لدى الاهل مثل الهزال او السمنه** |
| شديد النشاط🞎متوسط 🞎 خامل 🞎 | **مستوى النشاط خلال اليوم** |
| نمط متوسط الحركة🞎 نمط قليل الحركه 🞎  نمط شديد الحركة🞎 | **نمط الحياة اليومية لدى العائله** |
|  | **عدد الساعات اليومي على التلفاز او الالعاب الالكتروني او الموبايل** |
